# Case Report: Transarterial Chemoembolization in Combination With Tislelizumab Downstages Unresectable Hepatocellular Carcinoma Followed by Radical Salvage Resection

**DOI:** 10.3389/fonc.2021.667555

**Published:** 2021-03-29

**Authors:** Jiashuo Chao, Qi Zhu, Desheng Chen, Xiao An, Aiqun Liu, Fei Zhou, Lin Yuan, Zhaowen Wang, Hongcheng Sun

**Affiliations:** ^1^ Department of General Surgery, Shanghai Organ Transplantation Medical Center, Shanghai General Hospital, Shanghai Jiaotong University School of Medicine, Shanghai, China; ^2^ Department of Neoplasms and Interventional Radiology, Shanghai General Hospital, Shanghai Jiaotong University School of Medicine, Shanghai, China; ^3^ Department of Radiology, Shanghai General Hospital, Shanghai Jiaotong University School of Medicine, Shanghai, China; ^4^ Department of Oncology, Shanghai General Hospital, Shanghai Jiaotong University School of Medicine, Shanghai, China; ^5^ Department of Pathology, Shanghai General Hospital, Shanghai Jiaotong University School of Medicine, Shanghai, China

**Keywords:** hepatocellular carcinoma, transarterial chemoembolization, immune checkpoint inhibitor, tislelizumab, downstaging therapy, salvage resection

## Abstract

**Introduction:**

Transarterial chemoembolization (TACE) is inefficient at converting unresectable hepatocellular carcinoma (uHCC) to resectable. Treatment with immune checkpoint inhibitors (ICIs) is an emerging strategy for uHCC. Combined therapy of TACE with ICIs is considered to improve the therapeutic effect.

**Case presentation:**

A 45-year-old man was diagnosed with a bulky HCC under cirrhotic background without distant metastasis. Curative resection was infeasible, and TACE plus tislelizumab (an ICI targeting PD-1) was applied. The treatment course, starting from TACE and followed by tislelizumab one week later, was repeated every four weeks. After three courses, the tumor showed striking shrink in volume with complete radiological response, which permitted salvage resection. Notably, pathological examination found complete necrosis of the tumor with massive infiltration of lymphocytes in the tumor-nontumor interface and extensive granulomatous inflammation in the surrounding nontumor liver, indicating activated immune response synergistically caused by TACE with tislelizumab. The patient is now living well without tumor recurrence for 6 months after surgery.

**Conclusion:**

TACE in combination with tislelizumab may represent a potent strategy for uHCC. Data from randomized clinical trials are needed to assess its safety and effect in the setting of preoperative downstaging therapy.

## Introduction

Hepatocellular carcinoma (HCC) is one of the most common and lethal cancers worldwide. Surgical treatments with the purpose of complete eradication of HCC, such as liver resection, ablation and transplantation, provide HCC patients a chance to be cured (1). However, only around 30-40% of HCC patients globally who are diagnosed with early disease are eligible for these procedures, and the majority of HCC patients have intermediate or advanced tumors at initial diagnosis, thus have a poor prognosis due to palliative nature of available treatment modalities (2). Transarterial chemoembolization (TACE), which attacks tumor through arterial injection of anti-cancer drugs and embolizing agents, is currently the main treatment option for the patients with unresectable HCC (uHCC) (2, 3). TACE may downsize tumors and convert uHCC to resectable (4), and salvage resection following TACE can further benefit these patients (5–8). However, only 7-18% of uHCC patients could undergo salvage resection after TACE due to the low response rate of HCC to TACE (5–8). In recent years, the rationale for TACE in combination with immunological intervention for HCC has been accumulating. On the one hand, TACE kills HCC cells and causes tumor-associated antigen release, which boosts tumor-specific CD8^+^ T-cell responses (9). On the other hand, TACE increases programmed death receptor 1 (PD-1) and programmed death ligand 1 (PD-L1) expression in HCC, which may prohibit antitumor immunity (10). Hence, combined use of TACE with immune checkpoint inhibitors (ICIs) may represent a promising strategy for optimizing tumor response and allowing salvage resection. Tislelizumab is a humanized IgG4 anti-PD-1 monoclonal antibody developed as an immunotherapeutic, anti-neoplastic drug (11, 12). Results of phase 1/2 clinical trials in both global and Chinese patients have demonstrated antitumor activity of tislelizumab in HCC (11, 12). Here, we reported that a case with uHCC was downstaged by TACE combined with tislelizumab and successfully underwent radical salvage resection.

## Case Presentation

**Figure 1:**
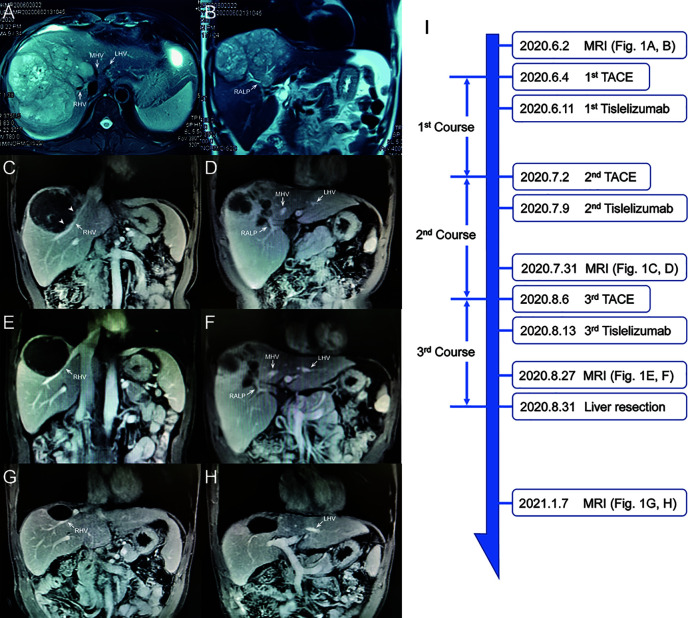
Abdominal MRI scan at different treatment phases. **(A, B)** Images at initial diagnosis. **(C, D)** Images after 2^nd^ course. Arrowheads indicate viable tumor lesions near the RHV. **(E, F)** Images before surgery. **(G, H)** Images at 4-month after surgery. **(I)** Timeline of treatment process. RHV, right hepatic vein; MHV, middle hepatic vein; LHV, left hepatic vein; RALP, right anterior lobe pedicle.

**Figure 2:**
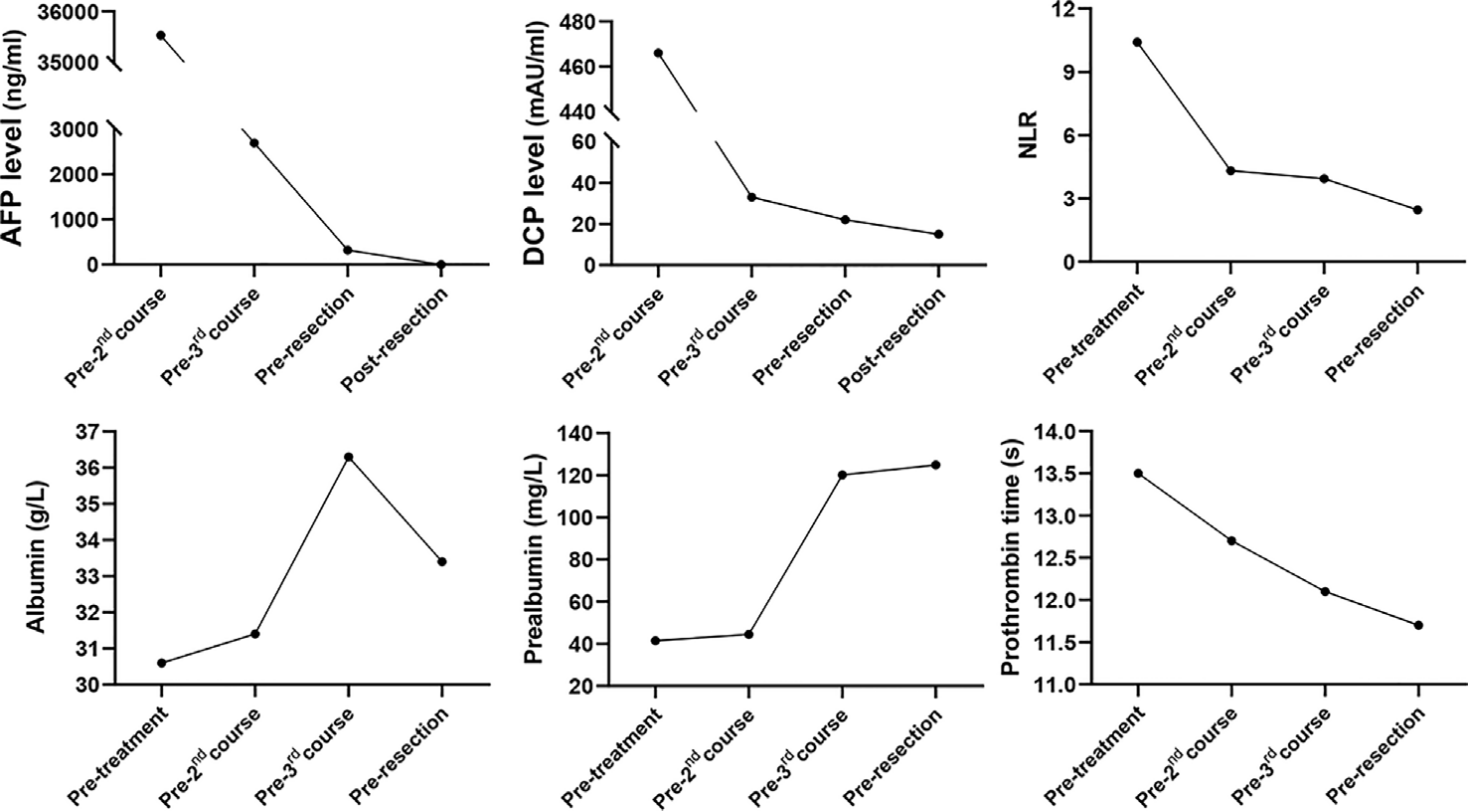
Dynamic changes in tumor marker levels, systemic inflammation index and liver function during treatment process. AFP, alpha-fetoprotein; DCP, des-γ-carboxy prothrombin; NLR, neutrophil-lymphocyte ratio.

**Figure 3:**
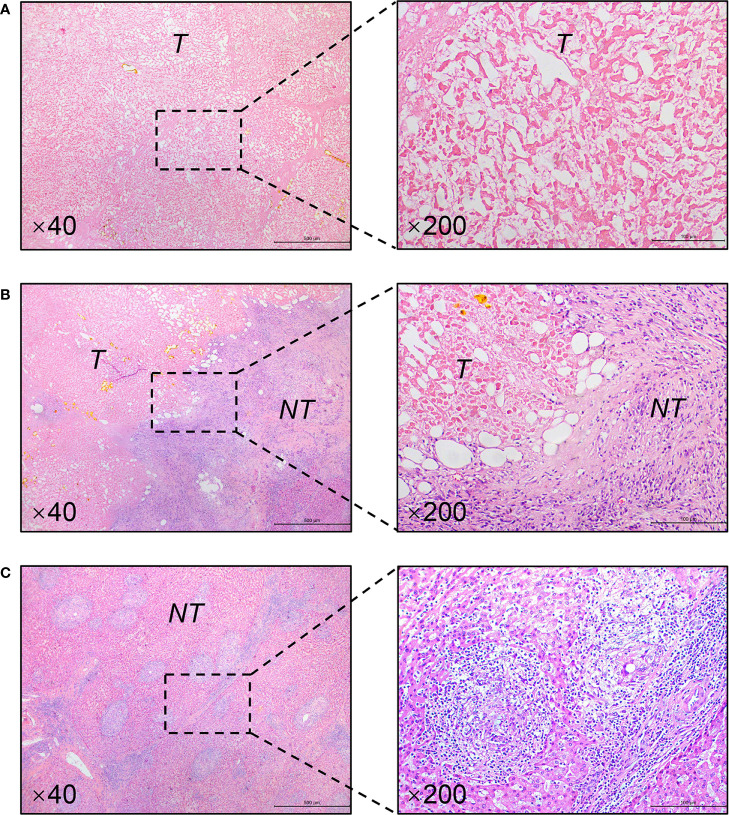
Representative pathological findings of resected specimens (H&E staining). **(A)** Complete necrosis of the tumor. **(B)** Massive infiltration of lymphocytes in the tumor-nontumor interface. **(C)** Typical granulomatous inflammation in the nontumor liver. Magnification is showed in the lower left corner. T, tumor; NT, nontumor liver.

The patient is a 45-year-old man with chronic hepatitis B virus (HBV) infection and liver cirrhosis. Contrast-enhanced magnetic resonance imaging (MRI) identified a huge irregular lesion with satellite nodules, around 15×12 cm in diameter, located in segment 7 (S7) and S8 (**Figures 1A, B**). The right hepatic vein (RHV) was surrounded by the tumor, and the middle hepatic vein (MHV) was closely attached to the tumor (**Figure 1A**). The right anterior lobe pedicle (RALP) was involved by the tumor near the first liver hilum (**Figure 1B**). Extrahepatic metastasis was not observed. Initial alpha-fetoprotein (AFP) level exceeded the detection limit of 1200 ng/ml. Considering the typical imaging characteristics of the tumor, high AFP level and HBV-related cirrhosis, the diagnosis of HCC was given without biopsy. Eastern Cooperative Oncology Group Performance Status (ECOG-PS) was 0, Child-Pugh score was 6 and Model for End Stage Liver Disease (MELD) score was 8. Consensus on the treatment was reached by a panel of experts from the multidisciplinary treatment (MDT) program. Radical resection was unattainable due to the bulky tumor and the cirrhotic background. After exclusion of contraindications, the patient received superselective TACE (oxaliplatin, epirubicin and iodized oil). And tislelizumab at a dose of 200 mg was infused intravenously one week later. TACE followed by tislelizumab was repeated every four weeks. Assessment of treatment response was based on Modified Response Evaluation Criteria in Solid Tumors (mRECIST) (13). After two treatment courses, MRI identified significant shrink and massive necrosis of the tumor (**Figures 1C, D**). Meanwhile, the MHV detached from the tumor, and the tumor-involved site of RALP departed from the first liver hilum. A relieved tumor load was reflected by the decreased levels of AFP, des-γ-carboxy prothrombin and neutrophil-lymphocyte ratio (**Figure 2**). Increased albumin and prealbumin levels as well as shortened prothrombin time indicated improved liver function (**Figure 2**). Nevertheless, the RHV was found adhered to the tumor and enhanced lesions still existed along the RHV, which indicated high risk of residual tumor cells along the vein once performing right anterior lobe resection (**Figure 1C**). Experts from MDT program assessed the situation and proposed associating liver partition and portal vein ligation for staged hepatectomy (ALPPS), but the patient refused to have operations twice. Therefore, we executed another treatment course of TACE plus tislelizumab. During the three courses, no immune-related adverse reaction (irAE) was observed. Two weeks after receiving tislelizumab, MRI revealed a complete radiological response (rCR) with clear tumor border and relieved tumor compression along the RHV (**Figures 1E, F**). The tumor was deemed resectable for the following reasons: (i) the origin of Glisson’s sheath of the right anterior lobe was visible (**Figure 1F**), which allowed precise ligation of the RALP without affecting blood inflow to S6; (ii) the presence of tumor capsule and rCR permitted a safe resection along the RHV, the outflow tract of S6 and S7. Radical resection was achieved by removal of S5, S8 and partial S7, and adequate future liver remnant (FLR) was ensured by preservation of S6 and partial S7. The patient recovered smoothly without any postoperative complications and discharged from hospital at 8^th^ day after surgery. Pathological examination reported two major lesions, 10 cm and 4.7 cm in diameter, with small satellite nodules. The tumor tissues showed complete necrosis, and the surrounding nontumor tissues presented massive infiltration of lymphocytes and extensive granulomatous inflammation (**Figure 3**). The patient was regularly followed up according to the standard protocol of our hospital. Four months after surgery, blood test showed normal AFP and des-γ-carboxy prothrombin levels and enhanced abdominal MRI revealed no sign of tumor recurrence in the liver (**Figures 1G, H**). The patient is now living well for more than 6 months without evidence of tumor relapse. A timeline of the treatment process was displayed in **Figure 1I**.

## Discussion

Salvage resection after TACE has yielded favorable outcome for patients with initially uHCC (5–7). However, salvage resection following TACE treatment can only be achieved in a small portion of the patients. The low conversion rate for salvage resection was largely attributed to unsatisfactory tumor response rate to TACE (14). To obtain better tumor response, TACE in combination with molecular targeted agents has drawn great attention (3). Previous clinical trials of combined TACE with tyrosine kinase inhibitors (e.g., sorafenib, brivanib and orantinib) failed to demonstrate better tumor response than TACE alone (15). In recent years, ICIs such as PD-1, PD-L1 and cytotoxic T-lymphocyte-associated protein 4 (CTLA-4) inhibitors have shown promising therapeutic effects for advanced HCC (16, 17). Although monotherapy with nivolumab or pembrolizumab in first- and second-line settings did not significantly improve survival outcomes in uHCC patients (16), combined regimen of atezolizumab plus bevacizumab was associated with better overall and progression-free survival than sorafenib (18). Data from a phase Ib study of pembrolizumab plus lenvatinib demonstrated a striking objective response rate (46.0%) superior to sorafenib (19). Accordingly, ICIs are increasingly considered as part of combination treatment, expected to provide synergistic effects against HCC. It’s rational to combine TACE with PD-1/PD-L1 inhibitor, because TACE could elicit PD-L1 expression in both intratumoral inflammatory cells and HCC cells, and PD-1 expression in inflammatory cells (10). Application of PD-1/PD-L1 inhibitors may enhance tumor response to TACE through inhibiting PD-1/PD-L1 signaling pathway in HCC. In this report, a bulky uHCC obtained rCR and became resectable after three courses of TACE plus tislelizumab. Furthermore, pathological examination revealed complete necrosis of the tumor (**Figure 3A**), which could not be easily achieved by TACE alone (**Supplementary Figure 1**). It suggests a potent antitumor activity of the combined regimen. To the best of our knowledge, this is the first report regarding combined use of locoregional therapy with ICIs in the preoperative setting.

Some questions still need to be clarified: (i) When to administer ICIs, during or after TACE? An ongoing trial (NCT04174781) is designed to simultaneously initiate TACE and PD-1 inhibitor in uHCC patients. Given that hepatotoxicity, an important irAE, occurs in up to 16% of patients receiving ICIs (20), attention should be paid to the safety issue regarding hepatotoxicity during concurrent use of TACE with ICIs. In our case, there was a one-week interval between TACE and tislelizumab, which may avoid superimposed hepatotoxic side effects. (ii) How many times of TACE are needed? TACE should be taken according to tumor response and follow an “on-demand” principle, and should not be continued if the tumor shows refractoriness (3). In our case, high fever (39°C) lasted for several days after 1^st^ and 2^nd^ sessions of TACE. We considered fever as therapeutic response to TACE reflecting tumor necrosis not injury to liver parenchyma, because TACE was performed with superselective technique to avoid collateral damage. Indeed, extensive necrosis of the tumor was observed after two treatment courses, which indicated decent response of the tumor to TACE. Mild fever (38.4°C) was presented with short duration after 3^rd^ session of TACE. (iii) Is salvage resection necessary after achieving rCR? Zhang et al. reported that salvage resection following TACE didn’t bring survival benefit to the uHCC patients who achieved rCR but improved the overall survival of patients with partial response (7). However, nine out of sixteen patients with rCR in that study were found to have residual viable tumor cells in resected specimens. Similarly, Shi et al. found the existence of viable tumor cells around necrotic tissues in two out of five patients whose tumors showed rCR after TACE (6). These data suggested that rCR didn’t represent complete eradication of tumors, and the patients with rCR may still bear high risk of tumor recurrence. In our case, though rCR was observed after 3^rd^ treatment course, high level of AFP (322 ng/ml) was also detected, indicating the possibility of residual tumor lesions. Thus, it’s necessary to carry out salvage surgery to completely remove the possible viable tumor cells. As expected, the AFP level returned to normal after surgery. (iv) What does granulomatous inflammation and lymphocyte infiltration indicate? Does TACE has synergistic effect with tislelizumab? The pathological examination of our case demonstrated extensive granulomatous inflammation in the nontumor liver and massive infiltration of lymphocytes in the tumor-nontumor interface, which were seldom found in HCC patients receiving TACE alone. We retrospectively inspected specimens from twelve patients with uHCC who received salvage resection after TACE, but didn’t observe massive lymphocyte infiltration or extensive granulomatous inflammation (**Supplementary Figure 1**). Besides, granulomatous inflammation is a rare finding in HCC patients receiving ICI treatment. Simoes et al. evaluated specimens from twenty HCC patients who received nivolumab treatment before surgery, and only one case showed granulomatous inflammation in the nontumor liver (the case also exhibited complete tumor necrosis) (21). We hold the view that the presence of massive lymphocyte infiltration and extensive granulomatous inflammation in our case may suggest activated immune response as a result of the synergetic effect of TACE plus tislelizumab. Nevertheless, some investigators deemed granulomatous inflammation as a manifestation of hepatotoxicity (20). Whereas, liver function of our patient was gradually improved during treatment process (**Figure 2**). A plausible explanation states that enhanced immune response, reflected by granulomatous inflammation and lymphocyte infiltration, destroys the tumor and thus improves liver function.

In summary, TACE in combination with ICIs may represent a potent therapeutic strategy for uHCC, especially as downstaging therapies. If tumors present favorable response and become technically resectable, salvage surgery should be taken into consideration to completely remove the tumors. Well-designed clinical trials are required to evaluate its efficiency and safety, and further demonstrate who may benefit from it.

## Data Availability Statement

The original contributions presented in the study are included in the article/**Supplementary Material**. Further inquiries can be directed to the corresponding author.

## Ethics Statement

Written informed consent was obtained from the individual(s) for the publication of any potentially identifiable images or data included in this article.

## Author Contributions

Conception and design: HS and ZW. Collection of clinical data and literature review: JC and DC. Administrative, technical, or material support (experts from the multidisciplinary treatment program; contributed clinical and pathological material; and discussed results and implications of findings): XA, AL, FZ, LY, ZW, and HS. Preparation of the manuscript: JC and QZ. Revision of the manuscript: HS and JC. All authors contributed to the article and approved the submitted version.

## Funding

This work was supported by the Clinical Research Innovation Plan of Shanghai General Hospital (CTCCR-2019C08) and the National Natural Science Foundation of China (81672846).

## Conflict of Interest

The authors declare that the research was conducted in the absence of any commercial or financial relationships that could be construed as a potential conflict of interest.
