# Supplementary material for: Case Report: Transarterial Chemoembolization in Combination With Tislelizumab Downstages Unresectable Hepatocellular Carcinoma Followed by Radical Salvage Resection
Source: Front Oncol. 2021 Mar 29;11:667555. doi: 10.3389/fonc.2021.667555 (PMC8044844; doi:10.3389/fonc.2021.667555)
Supplement: Supplementary file 1 [file Image_1.pdf]

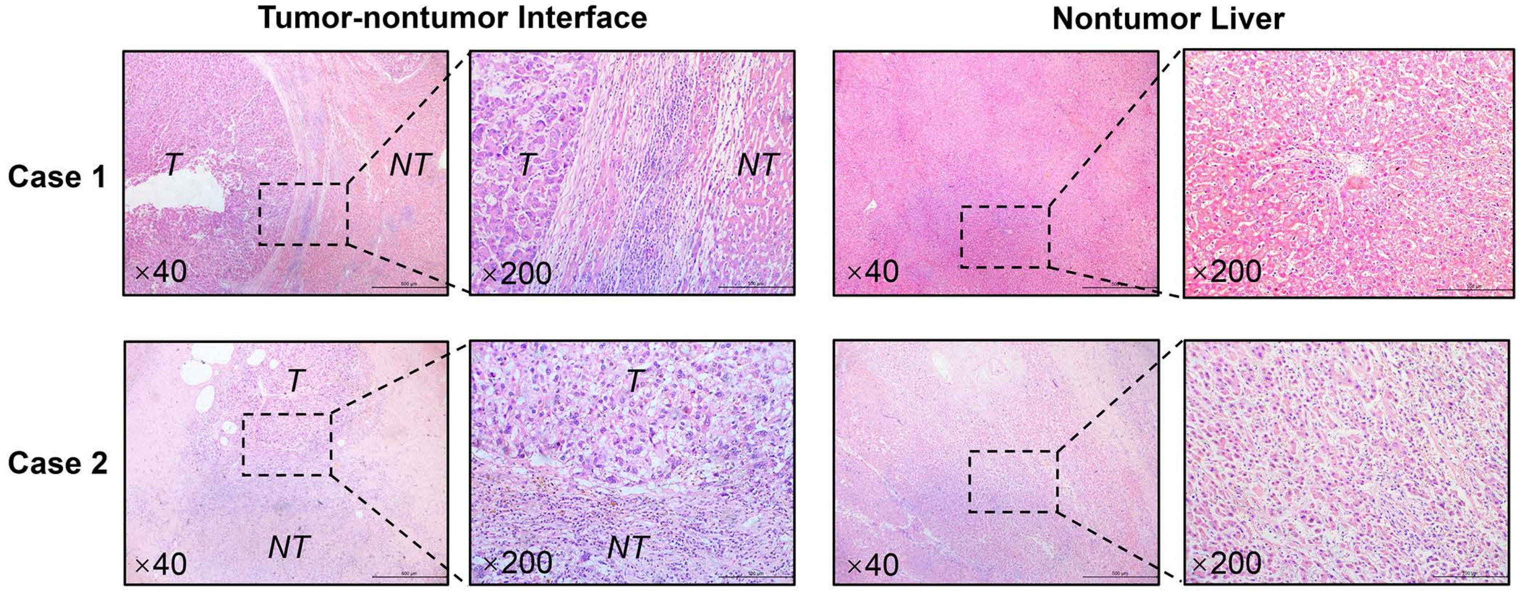

Supplementary Figure 1. Representative pathological findings of specimens from two of the twelve uHCC patients who received salvage resection after TACE (H&E staining). In the "tumor-nontumor interface" panel (left), viable tumor cells in the tumor side and mild lymphocyte infiltration in the nontumor side can be observed. In the "nontumor liver" panel (right), granulomatous inflammation can't be observed. Magnification is showed in the lower left corner. *Abbreviation:* T, tumor; NT, nontumor liver.
